# Biomedical evaluation of antioxidant properties of lamb meat enriched with iodine and selenium

**DOI:** 10.1515/biol-2022-0020

**Published:** 2022-03-17

**Authors:** Tatiana M. Giro, Sergey V. Kozlov, Ivan F. Gorlov, Andrey V. Kulikovskii, Anna V. Giro, Marina I. Slozhenkina, Dmiytiy V. Nikolaev, Alireza Seidavi, Alexander A. Mosolov

**Affiliations:** Department of Technology of Production and Processing of Livestock Products, N.I. Vavilov Saratov State Agrarian University, Teatralnaya Sq. 1, Saratov 410012, Russia; Department of Livestock Production, Volga Region Research Institute of Meat-and-milk Production and Processing, Volgograd, Russia; The Gorbatov’s All-Russian Meat Research Institute, Moscow, Russia; Department of Animal Science, Rasht Branch, Islamic Azad University, Rasht, Iran; Department Animal Diseases and Veterinary and Sanitary Examination, N.I. Vavilov Saratov State Agrarian University, Teatralnaya Sq. 1, Saratov 410012, Russia; Scientific Laboratory Department, Volga Region Research Institute of Meat-and-milk Production and Processing, Volgograd, Russia

**Keywords:** lamb, feed additives, essential microelements, bioorganic iodine and selenium, antioxidant properties, rat rations, acute toxic hepatitis

## Abstract

The article presents a study of the antioxidant properties of meat from lambs that received organic forms of iodine and selenium during growth. This meat was included in diets of laboratory animals using a model of acute toxic hepatitis. The experiments resulted in developing and testing a technique that was effective in enriching lamb with bioorganic elements of iodine and selenium and contributed to the activation metabolism in the bodies of animals consuming the meat. The purpose of the presented investigation was to compare the roles of bioorganic iodine and selenium and their combination as antioxidants in rat rations using a model of acute toxic hepatitis induced by carbon tetrachloride. The experimental studies have established a hepatoprotective effect of lamb meat enriched with selenium and iodine on rats suffering from toxic xenobiotic effects. This was confirmed by normalized hematological and biochemical measures in the blood of the experimental rats.

## 1 Introduction

According to the World Health Organization (WHO), the issue of iodine deficiency affects more than 2 billion people on the planet, with various pathologies being recorded against the background of thyroid gland disorders. More than one-third of them are known to suffer from endemic goiter; in 2%*t* of such cases, iodine deficiency leads to mental retardation. The deficiency of iodine also increases the oncological risk [1,2,3,4,5,6,7,8,9,10,11,12,13,14,15,16]. In the Russian Federation and in neighboring countries, there have been a substantial number of diseases reported to be associated with iodine deficiency. According to the WHO, adults need to consume about 150 µg/day of iodine, pregnant women at least 250 µg/day, and schoolchildren from 100 to 299 µg/day to prevent deficiency resulting in clinical abnormalities [17,18,19,20].

Moreover, iodine deficiency is known to be directly related to inadequate selenium intake. Selenium in rations causes a sharp decrease in the rate of chemical reactions in rats suffering from hepatic necrosis, primarily due to the effect of selenium on the metabolisms of leukotriene, thromboxane, and prostacyclin, which are lipoproteins. Furthermore, selenium and iodine in the ration influence humoral immunity [2,18,21,22,23,24,25,26,27,28]. Selenium-supplemented diets provide these effects on histological features of the liver and kidney in Tilapia [29]. According to Drutel et al. [30], a lack of selenium leads to a decrease in deiodinase activity and, subsequently, hypothyroidism. Besides, a number of selenoproteins (for instance, protein P) in blood plasma protect the body from free radical processes. Positive effects of selenium have been shown for the treatment of hepatitis C, cancer, cerebrovascular insufficiency, Alzheimer’s disease, poisoning with salts of heavy metals, thyroid disease, cardiovascular diseases, and asthma [30].

Consequently, fortification of food with these important microelements is an important problem; its accomplishment could improve the quality of human life.

One of the most important stages in the process of enriching products of animal origin with various microelements, including iodine and selenium, is feeding the animals with special feed additives. Feed additives containing iodine and selenium in animal rations promote the activation of metabolism, with iodine and selenium interacting with certain protein structures (deiodinases, glutathione peroxidases, and thioredoxin reductases) to form bioorganic iodine and selenium compounds. Such bioorganic forms of iodine and selenium have increased biological activity in contrast to the usual forms of mineral microelements and can be consumed in almost unlimited quantities without causing toxic effects due to their organic structures [6,7,8,11,17,18,19].

Currently, the *in vivo* development of qualitative characteristics of raw materials of animal origin is becoming one of the main tools for creating functional and even personalized nutrition [1,2,4,5,6,12]. The biofortification of meat composition is possible through the optimal amount of essential nutrients that can be introduced into rations at specified stages of growth [12,13,14,15].

There are many technologies to enrich meat with organic selenium, so the effectiveness of lamb fortification is of great interest. The main advantage of the additive for lamb is the low risk of a negative effect (e.g., overdosing) because the additive is already developed and approved for use in animal feed [27]. If the prescription and technology are followed precisely, mandatory control over the trace elements in the finished product is not required.

Thus, the search for ways and means of replenishing the iodine and selenium deficiency in human and animal nutrition is an urgent problem. Its solution will contribute to the prevention of pathologies caused by a deficiency of these microelements [30].

The objective of the research was to comparatively examine bioorganic iodine and selenium as antioxidants in rat rations using a model of acute toxic hepatitis induced by carbon tetrachloride (CCl_4_). The lamb was enriched with iodine and selenium through the introduction of selenium-iodine-containing feed additives (“DAFS – 25” and “Yoddar-Zn”) into the ram rations.

## 2 Materials and methods

To increase the nutritional value of feed, replenish the deficiency of macro-and micronutrients in the sheep body, and obtain products with a high biological value and a balanced chemical composition, we used feed additives “Yoddar-Zn” and “DAFS-25” based on organic silicon (Koretron) and a protein–carbohydrate component-cold-pressed pumpkin cake in a ratio of 1 kg/100 kg of the additive. These additives contain organic trace elements and minerals (Table 1).

**Table 1:** The composition of the feed additive based on “Yoddar-Zn,” plant silicon, and protein–carbohydrate complex

Ingredient	Amount
Plant silicon (diotomite “Koretron”)	1.0%
Iodine additive “Yoddar-Zn”	1.0%
Protein–carbohydrate complex	98.0%
Based on pumpkin cake	20.00

“Yoddar-Zn” is a source of bioavailable iodine in organic form and zinc. DAFS-25 is an organic substance – diacetophenonyl selenide (technical specifications 9337-001-26880895-96, certificate of state registration No. PVR 2.04.0185-96) with a weight fraction of selenium of at least 25% (Table 2).

**Table 2:** The composition of the feed additive based on DAFS-25, plant silicon, and protein–carbohydrate complex

Ingredient	Amount
Plant silicon (diotomite “Koretron”)	1.0%
Selenium additive DAFS-25	0.16 mg/100 g
Protein–carbohydrate complex	99.0%
Based on pumpkin cake	20.00

“Koretron” is a gray powder that consists of biogenic amorphous silica. This mineral additive is used in the production of mixed feed and premixes for various types of farm animals and poultry, acts as an anticaking agent and mycotoxin adsorbent, and is a source of water-soluble silicon (organic form) that is necessary for the stable functioning of the smooth muscles of the gastrointestinal tract of animals and poultry and improving calcium absorption [1,10].

The effectiveness of the feed additive was studied on young male sheep (ram lambs) of the Edilbay breed under conditions of the experimental livestock production enterprise of the N.I. Vavilov Saratov State Agrarian University.

Our research involved two stages. At the first stage, four groups of ram lambs aged 4 months with ten heads each were formed, including one control group and three test groups. Ram lambs received the general ration (GR) in the control group, GR with the feed additive “Yoddar-Zn” in group I, GR with the feed additive “DAFS 25” in group II, and GR with both feed additives “Yoddar-Zn” and “DAFS 25” in group III. Ram lambs were selected according to the analog pair method. All ram lambs received a GR; animals in test groups received the appropriate feed additives in an amount of 1% added to the GR.

Five ram lambs from each group were slaughtered. The carcasses were boned, the meat was chopped on a meat grinder plate, and a composite sample was prepared for further research.

At the second stage, five groups of white nonlinear rats were formed and fed with lamb meat from the initial trial to assess the antioxidant properties of iodine, selenium, and their combination. To set up the experiment, we used CCl_4_ as an effective agent to induce toxicity in the rats before the feeding trial began.

The oxidative stress caused by CCl_4_ is known to be aggravated by the suppressed activity of antioxidant enzymes and a decrease in the content of priority antioxidants, such as α-tocopherol and reduced form of ubiquinone in the cell.

In this regard, CCl_4_ is used in most modeling cases to assess *in vivo* the antioxidant properties of various biologically active compounds commonly applied as dietary additives for food or as components of functional food [28].

Microelement compositions were studied by atomic absorption spectrometry (GOST EN 31707-2012).

Organic iodine in forms of monoiodotyrosines and diiodotyrosines was determined by the HPLC with mass spectrometric detection in accordance with GOST 33422-2015. To determine the iodinated amino acids, we used the method of liquid quadrupole mass spectrometry with electrospray ionization. The studies were performed using an Agilent 1200 HPLC system with an Agilent 6410 mass spectrometric detector (Sciex Exion LC HPLC system) – “SCIEX CORPORATION”, Singapore. The sample preparation included hydrolysis with proteolytic enzymes and purification of the hydrolyzate by the solid phase extraction (SPE). Before the instrument identification, precolumn derivatization with butanol and acetyl chloride was carried out [26].

The organic form of selenium was determined by an innovative MI-06-2021 method “Determination of selenium in organic form using tandem liquid mass spectrometry” developed at the Gorbatov’s All-Russian meat research institute. The method of liquid quadrupole mass spectrometry with electrospray ionization was used to determine the selenium-containing amino acid selenomethionine. The studies were carried out on a Sciex Exion LC HPLC system with a Sciex QTRAP 5500 hybrid mass spectrometer (“SCIEX CORPORATION”, Singapore) with an activated ion trap. The sample preparation included hydrolysis with proteolytic enzymes and purification of the hydrolyzate by SPE. Precolumn derivation was performed before the instrumental identification.

The toxic CCl_4_ effect is known to be primarily associated with the prooxidant effect of free radicals formed during its metabolism – trichloromethyl {\text{CCL}}_{3}^{\ast }] and highly reactive trichloromethylperoxyl CCl_3_OO* [28]. The interacting CCl_4_ radicals and polyunsaturated fatty acids of membrane phospholipids initiated lipid peroxidation (LPO) with the subsequent development of a chain reaction of free radical oxidation. This leads to a profound disruption of the functional properties of membranes, such as suppression of activity of membrane-bound enzymes, release of cytosolic enzymes into the blood, and, ultimately, apoptosis and necrosis of hepatocytes.

To determine antioxidant properties of the feed additives under study, five groups of white nonlinear rats were formed. General principles of the experimental design are presented in Table 3.

**Table 3:** Study design using white nonpedigree rats as experimental animals

Time	Group 1, *n* = 8 (background)	Group 2, *n* = 7		Group 3, *n* = 7		Group 4, *n* = 8	Group 5, *n* = 7 (positive control)
Start	Weighing of animals	Weighing of animals. Introduction of trichloromethane in olive oil, ratio of 50:50, volume of 2 mL/kg					
Day 2	Weighing of animals						
	GR + pure meat	GR + meat obtained from ram lambs fed with “Yoddar-Zn”	GR + meat obtained from ram lambs fed with DAFS-25		GR + meat obtained from ram lambs fed with “Yoddar-Zn” and “DAFS-25”		GR + pure meat
	Clinical study of animals. Weighing. Aspiration of blood for hematological and biochemical studies						
Day 7	Clinical study of animals. Weighing. Aspiration of blood for hematological and biochemical studies						
Day 14	Clinical study of animals. Weighing. Aspiration of blood for hematological and biochemical studies						

Note: GR is the general ration of experimental animals (white nonpedigree rats).

The study applied a method of analogs, so there were formed the following groups of rats:

− Control group I (*n* = 10): rats were fed with a GR added with lamb meat from ram lambs raised on a standard diet used on the farm;
− Test group II (*n* = 10): rats suffering from CCl_4_-induced toxic hepatitis were fed with a GR added with lamb meat from ram lambs that received “Yoddar-Zn” as a feed additive;
− Test group III (*n* = 10): rats suffering from CCl_4_-induced toxic hepatitis were fed with a GR added with lamb meat from ram lambs that received “DAFS-25” as a feed additive;
− Test group IV (*n* = 10): rats suffering from CCl_4_-induced toxic hepatitis were fed with a GR added with lamb meat from ram lambs that received “Yoddar-Zn” and “DAFS-25” feed additives in combination; and
− Control group V (positive control; *n* = 10): rats suffering from CCl_4_-induced toxic hepatitis were fed with a GR added with lamb from rams grown on a standard diet used on the farm.

White nonpedigree rats were selected for the experiment according to the “random numbers” method, with their body weight being considered as a determining criterion. Individual values of body weights did not deviate from the average value in the group by more than 10%. The rats were weighed on a PA2102C precision balance (OHAUS, Switzerland).

Throughout the experiment, clinical states of the rams, their activities, and consumption of food and water were recorded.

For hematological and biochemical studies, blood was collected from the tail vein from five random rats in each group. Previously, the skin was disinfected with a 70% solution of ethyl alcohol along the vein. Plasma and serum of rats were examined on days 2, 7, and 14 after the beginning of the experiment.

The biochemical analyses of blood samples were conducted in vacuum tubes for *in vitro* diagnostics “Improvacuter” (Guangzhou Improve Medical Instruments Co. Ltd, China), using thrombin as a clot activator, 2 mL each. For hematological measurements, we applied 0.1–0.2 mL of thrombin in microtubes with K2 EDTA anticoagulant for capillary plasma of 200 µL, “UNIVET” in the modification “UNIVET-Pm,” and specifications 9398-033-59879815-2012.

The study of the morphological composition of peripheral blood considered the numbers of erythrocytes, leukocytes, and platelets, as well as the hemoglobin level. To determine the systemic effect, we recorded main parameters of metabolism in the serum – total protein, albumin, urea, creatinine, glucose, bilirubin, as well as the activities of alkaline phosphatase, aspartate, and alanine aminotransferases. The biochemical studies were performed using a “StatFax 3300” biochemical analyzer (Awareness Technology, USA) using the “Deacon DS” diagnostic systems. To check the correctness and accuracy of the biochemical parameters determined in the serum, we used the control serum, according to the specifications 9398-022-09807247-2009, HOSPITEX DIAGNOSTICS LLC.

The results obtained were statistically processed according to the standard procedures using Microsoft Excel 2016 (Microsoft Corp., USA), StatPlus 2009 Professional 5.8.4 for Windows (StatSoftInc., USA), and Student’s *t*-test to assess the significance of differences between samples of test and control experiments. The mean absolute error and standard deviation calculated for this sample enabled determining the standard arithmetic mean error and its confidence limit considering Student’s *t*-coefficient (*n*, *p*) at a significance of 95% (*p* = 0.05) and the number of measurements of *n* = 5.

The significance of the mean values in the test and control experiments was evaluated by the *p*-value in the variant of the two-sample unpaired *t*-test with unequal variances.

Differences were considered significant when *p* ≤ 0.05. Moreover, there was observed the inequality *t*, *t* (*n*, *p*) at *n* = (df + 1), where df is the variance, *p* = 0.05, where *t* = |*x*1 − *x*2|/(*s*12 + *s*22)1/2, *x*1 and *x*2 were arithmetic mean values; *s*12 and *s*22 were their standard errors for two samples of experimental data.

Digital material is presented in SI units recommended by the WHO and the CMEA standard 1062-78.


**Ethical approval:** The research related to animal use has been complied with all the relevant national regulations and institutional policies for the care and use of animals. The studies were performed in the vivarium of the N.I. Vavilov Saratov State Agrarian University. The experiments were carried out in accordance with the “Rules of laboratory practice in the Russian Federation” (Order of the Ministry of Health of the Russian Federation No. 708n dated 08.23.2010) and methodological instructions with the “Guidelines for conducting preclinical studies of drugs. Part one” (2012). The experiments on animals fully complied with the rules adopted by the European Convention for the Protection of Vertebrate Animals Used for Experimental and other Scientific Purposes (ETS 123) Strasbourg, 1986.

## 3 Results and discussion

The trace element compositions of lamb meat from ram lambs at the age of 7 months after being fed with different rations are presented in Table 4.

**Table 4:** The concentration of trace elements in lamb from experimental rams, µg/kg*,**

Parameter	Group			
	Control	Test I	Test II	Test III
Aluminum (Al)	0.55 ± 0.044	0.54 ± 0.044	0.54 ± 0.06	0.53 ± 0.06
Iodine(I), total content [µg/kg]	31.8 ± 2.4	35.0 ± 2.4	54.6 ± 6.8***	61.5 ± 9.6***
Silicon (Si)	26.28 ± 2.61	29.26 ± 2.41	29.75 ± 2.91	30.75 ± 2.91
Selenium (Se) [µg/kg]	199.7 ± 69.9	286.3 ± 100.2	253.4 ± 88.7	300.2 ± 105.1
Zinc (Zn)	31.15 ± 2.21	31.15 ± 2.21	36.54 ± 3.17	36.54 ± 3.17

Note: hereinafter, control group and test group(s) are compared *p ≤ 0.05; **p ≤ 0.01; and ***p ≤ 0.001.

The highest iodine contents of 54.6 ± 6.8 and 61.5 ± 9.6 µg/kg were found in test groups II (“Yoddar-Zn” in the ram lamb diet) and III, respectively, which were 1.7 and 1.9 times more than in the control group.

Our studies of the antioxidant properties of organic forms of iodine, selenium, and their combination in the rat model of acute toxic hepatitis showed the clinical symptoms of intoxication that developed on the second day after the xenobiotic application. The animals were depressed, inactive, or even lying down. The hair was tousled without a characteristic shine, the mucous membranes and skin were pale with a yellowish tinge, and the appetite was reduced.

Remission of the rats’ general condition was noted on the 7th day of the experiment in groups II, III, and IV. The rats became more active in comparison with the rats in the control group, took food, made movements, and reacted to external stimuli.

An improvement in the general condition of rats in test group V was recorded only on the 14th day.

On the 14th day, the rats in test group IV had no clinical symptoms of intoxication, whereas most animals in test groups II and III showed slight hypodynamia and tousled hair that were recorded up to 18th day after the start of the experiment.

The deaths of rats were noted in test groups I, II, and III on the 8th day, one individual in each group. Two rats died on the 6th and 8th days of the experiment in group V (positive control).

In the control group (background), no signs of intoxication were observed.

**Figure 1:**
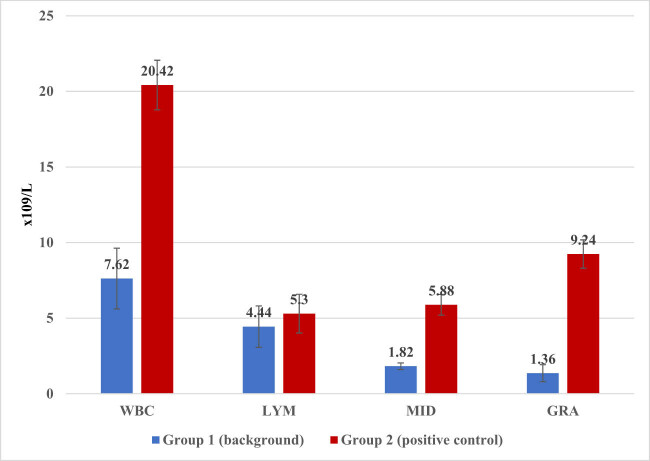
The dynamics of leukocytes 24 h after the injection of CCl_4_ (WBC: white blood cells, LYM: lymphocytes, MID: content of a mixture of monocytes, eosinophils, basophils, and immature cells, and GRA: granulocytic cells).

Two days after the start of the experiment, the hematological analysis of the peripheral blood of test rats (Figure 1) showed a significant increase in the total number of leukocytes in rats suffering from CCl_4_-induced hepatitis compared with the rats in the control group. An increase in the total number of leukocytes occurred due to granulocytic cells, which confirmed the development of inflammatory processes in the body of rats, in particular in the liver affected by CCl_4_.

The biochemical analysis of rat blood serum (Table 5) found a significant increase in liver enzymes of alanine and aspartic 24 h after the injection of a 50% solution of CCl_4_ in olive oil.

**Table 5:** The dynamics of biochemical measures in animal blood 24 h after the start of the experiment (*n* = 5)*

Parameter	Unit of measurement	Group I (background)	Group V (positive control)
		*M* ± *m*	*M* ± *m*
ALT	E/L	63.7 ± 18.67***	936.0 ± 106.3
AST	E/L	48.64 ± 3.04***	844.0 ± 111.28
Glucose	Mmol/L	6.17 ± 1.99	2.07 ± 0.7
Total protein	g/L	58.06 ± 3.04	54.38 ± 6.83
Albumin	g/L	32.56 ± 1.69	29.88 ± 3.7
Globulin	g/L	25.5 ± 2.35	24.5 ± 5.25
Alkaline phosphatase	E/L	290.7 ± 62.50**	502.0 ± 6.1
Bilirubin	µmol/L	0.13 ± 0.08***	2.95 ± 0.32
Urea	mmol/L	6.9 ± 0.31	6.4 ± 1.9
Creatinine	µmol/L	43.18 ± 4.45	44.80 ± 3.45
Cholesterol	mmol/L	2.0 ± 0.002	2.0 ± 1.0

Note: hereinafter, control group and test group(s) are compared *p ≤ 0.05; **p ≤ 0.01; and ***p ≤ 0.001.

**Figure 2:**
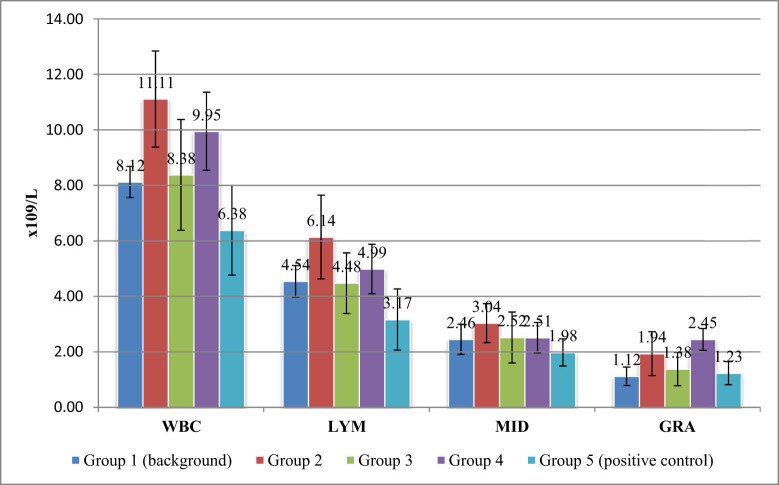
The dynamics of peripheral blood leukocytes in rats (WBC: white blood cells; LYM: lymphocytes; MID: content of a mixture of monocytes, eosinophils, basophils and immature cells; and GRA: granulocytic cells).

The hematological analysis at the end of the experiment found a significant decrease in the total number of leukocytes (*p* = 0.0236) in group V rats that were injected with CCl_4_ without therapeutic measures compared with the control rats, which indicated posttraumatic depletion of the immune system, whereas the number of leukocytes of rats in test groups II and III that received lamb enriched with selenium and iodine, respectively, was slightly higher than in the control group (background; Figure 2).

The leukocytes values in test group III rats fed with the dietary additive based on iodine practically did not differ from those in the control (background) group. This fact was considered as the antioxidant effect of selenium that prevented the accumulation of LPO products and contributed to the reduction of glutathione and apoptosis of liver cell elements that were utilized by leukocytes [6].

The higher contents of active bioorganic iodine and selenium in comparison with control ram lambs were due to an increase in the amount of selenium-dependent enzyme glutathione peroxidase that enhanced the antioxidant effect. In general, the glutathione peroxidase enzyme consists of several related groups of enzymes that are synthesized in different cells of a living organism [6,7,8,11,17,18,19,26,31].

The biochemical measures of the rats’ serum at the end of the experiment indicated the restoration of the structural and functional properties of hepatocytes and a decrease in inflammatory and destructive processes in the liver because a significant decrease in indicator hepatic enzymes (AST and ALT) was recorded in rats in all groups (Table 6).

**Table 6:** The biochemical parameters of rat blood serum*,***

Parameter	ALT (E/L)	AST (E/L)	Glucose (Mmol/L)	Total protein (g/L)	Albumin (g/L)	Globulin (g/L)
Group I (background)	61.6 ± 2.7	42.9 ± 4.0	6.5 ± 1.5	67.2 ± 5.1	32.7 ± 3.2	34.5 ± 3.8
Group II	60.3 ± 1.5	76.8 ± 5.3	5.4 ± 1.5	56.9 ± 3.6	24.8 ± 4.03	32.1 ± 5
Group III	49.4 ± 2.9**	73.4 ± 3.3	6.7 ± 3.7	95.8 ± 2.5	52.5 ± 14.5	43.2 ± 1.3
Group IV	65.2 ± 4.8	43.4 ± 1.9	5.9 ± 1.3	65.7 ± 3.9	32.5 ± 2.1	33.2 ± 1.7
Group V (positive control)	64.5 ± 2.0	75 ± 4.7	5.3 ± 1.6	64.9 ± 6.0	20.2 ± 2	44.7 ± 5.0

Note: hereinafter, control group and test group(s) are compared *p ≤ 0.05; **p ≤ 0.01; and ***p ≤ 0.001.

High activity of aspartate aminotransferase against the background of high concentrations of albumins and carbohydrates indicated an increase in the energy metabolism in the animal body as transaminases provided a relationship between the metabolisms of nitrogenous compounds and carbohydrates in the animal body. An increase in globulins may be a consequence of an increase in the monocytic-macrophage function of the liver, which followed from the iodine effect on the animal organism [30].

There was no difference between the indices of biochemical parameters and blood serum and background values in the test group IV rats fed with lamb enriched with selenium and iodine. This fact can be explained as a consequence of the synergism of iodine and selenium that contribute to an increase in the body’s compensatory factors under the action of xenobiotics [32].

The decrease in the albuminsynthesizing function of the rat’s liver against the xenobiotics as well as increase in globulin fractions is a consequence of the release of proteins into the bloodstream in the acute phase of inflammation, which indicates inflammatory and destructive processes in the liver parenchyma [12]. In our opinion, these indices were due to an increase in energy consumption to restore the functional activity of hepatocytes damaged by xenobiotics [12].

Thus, the presented data allowed us to claim that selenium and iodine enriched lamb in the diets of rats suffering from toxic hepatitis caused by xenobiotic helped to restore the functioning of the liver parenchyma cells (hepatocytes) in rats [17,18,19,26].

## 4 Conclusion

The production of domestic meat raw materials enriched with organic microelements is a promising technology for functional nutritional products. During the research, the form of organic iodine was reliably identified, and its amount in the form of iodotyrosines was monitored. The ability of the organic form of iodine to exhibit various biological properties, in particular, through iodine-containing hormones, such as thyroxine and triiodothyronine, participating in the regulation of all metabolic processes was documented with potential relevance in humans. The presence of iodotyrosines in lamb from ram lambs fed with the special additives was confirmed, and their iodization degree was determined. The feed additives with organic forms of iodine and selenium in the ration of small ruminants make it possible to obtain lamb enriched with bioavailable microelements, which has the potential of medical and social importance for the prevention of iodine deficiency, enhancement of immunity, and normalization of metabolism and the endocrine system.

A pronounced antioxidant effect of the studied drugs was established. The hematological studies were conducted, and a decrease in the total number of leukocytes was found in rats injected with a toxicant without therapeutic measures compared with the control group rats. Changes in the number of leukocytes in the peripheral blood of rats were studied; an increase in the activity of aspartate aminotransferase was proved in the group of rats that consumed lamb enriched with iodine. Differences in biochemical parameters (ALT, AST, glucose, total protein, albumin, globulin, alkaline phosphatase, bilirubin, urea, creatinine, and cholesterol) of the rat blood serum were found; positive synergistic effects of iodine and selenium that promoted an increase in the compensatory factors of the organism on xenobiotic action were noted. Considering that most pathologies caused by micronutrient deficiency lead to the accumulation of free radicals and further exacerbate the pathological process, we expect that enriched products in rations will help stop the destructive inflammatory processes against the background of the primary treatment.

Lambs whose muscle tissues were enriched with the above feed additives individually or in combination and introduced into the diet of rats contributed to an increase in the general resistance of the organism and, as a consequence, the prevention of many pathologies of infectious and noninfectious etiology.
